# Simultaneous adsorption of Cu(II), Zn(II), Cd(II) and Pb(II) from synthetic wastewater using NaP and LTA zeolites prepared from biomass fly ash

**DOI:** 10.1016/j.heliyon.2023.e20253

**Published:** 2023-09-21

**Authors:** Mehmet Emin Küçük, Iryna Makarava, Teemu Kinnarinen, Antti Häkkinen

**Affiliations:** a Department of Separation Science, School of Engineering Science, Lappeenranta-Lahti University of Technology LUT, Opistognathus 34, FI-53850, Lappeenranta, Finland; b Hydrometallurgy and Corrosion, Department of Chemical and Metallurgical Engineering (CMET), School of Chemical Engineering, Aalto University, P.O. Box 16200, FI-00076, Espoo, Finland

**Keywords:** Zeolite synthesis, Biomass ash, Adsorption, Isotherm, Kinetics

## Abstract

Herein, NaP and LTA zeolites were successfully synthesised from woody biomass ash with alkali fusion-assisted hydrothermal method by altering the NaOH/ash ratio, crystallisation time and crystallisation temperature. In order to reduce the synthesis costs, NaP zeolite was synthesised with no additional source of aluminium and silicon. The synthesised zeolites were utilized for the monocomponent and simultaneous adsorption of Cu(II), Cd(II), Pb(II) and Zn(II) ions. The maximum adsorbed amount of metals had the trend Pb(II) > Cu(II) > Cd(II) > Zn(II) for both NaP and LTA zeolite. The kinetic data fit well to the pseudo-second order model indicating that chemisorption is the rate-limiting step. The isotherm data were well described with Sips and Redlich-Peterson models indicating a non-ideal heterogeneous adsorption process. Maximum adsorption capacity of NaP zeolite was 42.9 mg/g for Cu(II) and 117.3 mg/g for Cd(II), while LTA had 140.1 mg/g and 223.5 mg/g for Cu(II) and Cd(II) ions, respectively.

## Highlights

• Successful synthesis of NaP and LTA zeolite performed from biomass ash.
• No additional Al or Si source was used for NaP synthesis.
• Sorption capacity of NaP is 42.9 and 117 mg/g for Cu(II) and Cd(II), respectively.
• Sorption capacity of LTA is 140 and 223 mg/g for Cu(II) and Cd(II), respectively.
• Affinity of NaP and LTA has the trend Pb(II) > Cu(II) > Cd(II) > Zn(II).

## Graphical abstract

**Figure:**
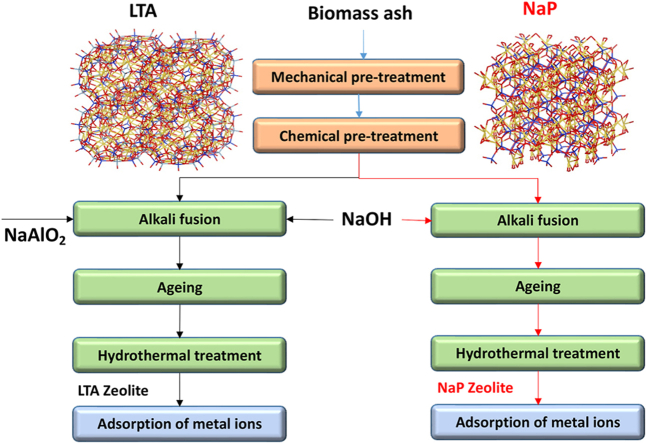

## 1 Introduction

Water pollution by discharge of hazardous metals has been one of the most serious problems threatening the human health and ecological system. The release of toxic metals may occur naturally (leaching of ore deposits, volcanic activities) or as a result of anthropogenic factors (mining and smelting processes, battery and machinery manufacturing, electroplating plants) [1]. Both Cd(II) and Pb(II) are extremely toxic metals that may form complexes in the liver and kidney, resulting in endocrine disorders, and cancer [2,3]. In addition, Cu(II) is responsible for problems in central nervous system, liver and kidney [4,5]. Thus, the European Commission has restricted the concentrations for Cu(II), Cd(II) and Pb(II) in drinking water to below 2.0, 0.005, and 0.005 mg/L, respectively [6].

Zeolites are a group of crystalline aluminosilicate frameworks with three-dimensional networks of corner-sharing tetrahedra (TO_4_, T: Al or Si) linked by oxygen atoms. In presence of Al^3+^, a negatively charged framework is formed, and an exchangeable alkaline or alkaline earth metal is bound to the structure [7]. This bonding leads to a framework with exceptional cation exchange characteristics [8]. Over 200 different types of zeolites have been synthesised from various raw materials including kaolinitic rock [9], coal fly ash [1], coal gangue [10], bagasse ash [11], peat ash [12], and alum sludge [13].

NaP zeolite (Zeolite P) is the synthetic counterpart of gismondite (GIS), with a general formula of M_2/n_O·Al_2_O_3_·1.8–5.0SiO_2_·5H_2_O, where M is an n-valent cation (e.g., Na, K). NaP has a two-dimensional pore system, with eight-ring intersecting channels of 0.26 nm × 0.59 nm and 0.31 nm × 0.44 nm in the lattice planes of [010] and [100], respectively. It has smaller micropore sizes (∼2.9 Å) compared to faujasite (FAU) (∼7.4 Å), MFI zeolite (∼5.4 Å), and LTA zeolite (∼4.1 Å) which makes it suitable for the separation of small molecules [1,14].

LTA zeolite (NaA) has the classical formula of M_2/n_O·Al_2_O_3_·2SiO_2_·4.5H_2_O, and it has a three-dimensional eight-ring framework that can be built by linking double sodalite (Na_8_(Al_6_Si_6_O_24_)Cl_2_) cages which create an alpha cavity in the middle of the structure [15]. There are three exchangeable cation sites in the framework, which are centred on the six rings of sodalite cages, near the centre of the eight rings around the alpha cavity and centred on the four-rings which can carry 8, 3, and 12 cations per cavity, respectively [16].

The presence of Al- and Si- bearing minerals makes biomass ashes favourable raw materials for zeolite synthesis. Nevertheless, the synthesis efficiency is hindered due to large impurity contents leading to a significantly fewer studies investigating their application compared to coal fly ash. Oliveira et al. [11] removed the unburnt organic matter in sugarcane bagasse ash by calcination at 600 °C for 8 h. The treated ash was used for the synthesis of LTA by the application of C_9_H_21_O_3_Al as the aluminium source. The synthesised zeolite had a Si/Al ratio of 1.71, and a maximum adsorption capacity of 142 mg Cu^2+^/g. Joseph et al. [12] prepared GIS-, LTA- and FAU-type zeolites from peat ash. After the removal of the organic matter through calcination, the remaining impurities were separated by leaching in concentrated HCl and HNO_3_ solutions. The Si/Al ratio was adjusted with addition of NaAlO_2_ as aluminium source. The prepared LTA and FAU zeolites were successful to remove cobalt, lead, cadmium, zinc, and copper from aqueous solution. In another extensive study investigating the synthesis parameters of NaP zeolite, the soluble compounds of fly ash were removed with water dissolution under mechanical stirring at room temperature. The solid product subsequently went through washing, filtration, drying, sieving and alkaline hydrothermal treatment step which was performed in an autoclave with 2 N NaOH solution at 150 °C. The prepared NaP had a specific surface area of 52.4 m^2^/g, and maximum adsorption capacity of 20.9, 7.9, 26.9 and 88.3 mg/g for Cu(II), Zn(II), Cd(II), and Pb(II), respectively [17]. For investigating the synthesis parameters of NaP zeolite (GIS), Zhang et al. [1] extracted the silica and alumina in coal fly ash by performing an acid extraction followed by the calcination of the ash with Na_2_CO_3,_ and precipitation of Si and Al in the form of Na_2_SiO_3_ and Al(OH)_3_. The prepared NaP zeolite with the addition of stearic hindrance agent cyclohexanol had a specific surface area of 80.3 m^2^/g and adsorption capacity of 39.96 mg Zn^2+^/g.

Although extensive research has been carried out on the application of coal fly ash, very little is known about the use of woody biomass ash for zeolite synthesis. Furthermore, its increasing annual production has heightened the need for more successful applications that targets its conversion into products with economical value. These inexpensive by-products play a critical role to reduce the zeolite synthesis costs in addition to their contribution in circular economy through reuse of secondary raw materials. This study provides new insights for the utilisation of woody biomass ash for zeolite synthesis through an efficient and inexpensive route, and it has set out to investigate 1) its single stage purification, and transformation to NaP zeolite without the use of any organic template and source of aluminium or silica, 2) synthesis of LTA with high purity, 3) the influence of the synthesis conditions including the NaOH/ash ratio, crystallisation temperature, and time on zeolite characteristics and 4) the removal of Cu(II), Zn(II), Cd(II), and Pb(II) from aqueous solutions by using the prepared zeolites. For a better investigation of the adsorption characteristics, the influence of adsorbent dose and pH of the effluent were studied in addition to adsorption kinetics and isotherm studies.

## 2 Experimental

### 2.1 Materials

The biomass fly ash (BA) was collected from a co-incineration plant of a paper mill located in Southeast Finland where bark, natural gas, wastewater sludge, wood chips and recycled wood are utilized as the fuel source. All chemicals HCl, NaOH, Cu(NO_3_)_2_∙3H_2_O, Zn(NO_3_)_2_, Cd(NO_3_)_2_∙4H_2_O, Pb(NO_3_)_2_, and NaAlO_2_ were purchased from Sigma-Aldrich and were used as received. Ultra-pure deionised water (Merck Millipore Q-POD, DI, 15 MΩ) was used throughout the experiments.

### 2.2 Characterisation

The chemical composition of the ash obtained with XRF analysis is presented in Table 1. The pH of the solutions during acid dissolution was monitored using a Radiometer PHM 240 pH/ion pH meter. Sample weights were measured using a Mettler AC 88 analytical balance. Characterisation of surface morphology of the ash samples and zeolites was revealed with a scanning electron microscope (Hitachi SU 3500 SEM, Japan). The elemental compositions of the samples were obtained with energy-dispersive X-ray spectroscopy (Thermo Scientific Ultradry EDS detector, USA), and the results were presented as the average of 3 measurements. An X-Ray fluorescence (XRF) spectrophotometer was utilized to obtain the elemental composition of the samples as oxides (Bruker AXS S4 Pioneer, USA). The XRF analysis was performed according to the glass fusion technique after grinding of the ashes. The compositions of the samples were determined according to the Wroxi method, which was developed by PanAnalytical and validated by the application of international Certified Reference Materials (CRM). The chemical composition and crystal structure of zeolites were recorded with an X-Ray diffractometer (XRD) at 40 Kv and 40 mA, with Cu Kα radiation, at 0.02 s/step over a 2θ range from 5° to 60° (Bruker D8 Advance X-Ray diffractometer, USA). The specific surface area (SSA), pore volume, and pore size of the samples were obtained with the N_2_ adsorption-desorption measurements at −196 °C (Micromeritics 3Flex, USA). The samples were dried in a vacuum oven (Memmert, Germany) at 200 °C at 100 mbar for 12 h, which was followed by their degas at 200 °C for 6 h prior to the analysis. Brunauer-Emmett-Teller (BET) model was used to calculate the surface area and micropore volumes were obtained with t-plot method. Characteristics vibration bonds of the samples between the wavelengths 400-4000 cm^−1^ were recorded via Fourier infrared spectrometry (FT-IR, PerkinElmer, UK). Zeta potential measurement of synthesised zeolites was performed by a Zetasizer ZS Nano (Malvern, UK) analyser. Zeolites were thoroughly washed, filtered, and dried. Electrophoretic light scattering method was utilized for zeta (ζ-) potential measurement according to methodology developed by Malvern Instruments using the surface zeta potential cell (Zen1020, Malvern Instruments) and the zeta potential transfer standard (DTS1235, Malvern Instruments) as tracer particle (zeta potential −42.0 mV ± 4.2 mV). Smoluchowski model was used to calculate ζ-potential values of particles in aqueous media (0.02 g zeolite in 100 mL water). pH adjustment was performed with the addition of NaOH and HCl droplets in the solution. Metal concentrations in the solutions before and after adsorption tests were quantified by inductively coupled plasma-mass spectroscopy (ICP-MS, Agilent 7900, USA) after their dilution in an acid mixture of 1% HCl and 1% HNO_3_. Relative standard deviation of all ICP measurements were less than 3.6%.

**Table 1:** The main components of BA obtained with XRF analysis; values expressed in wt.%.

Composition	CaO	SiO_2_	Al_2_O_3_	SO_3_	Fe_2_O_3_	MgO	Na_2_O	TiO_2_	Others	LOI_950°C_
wt. (%)	31.49	27.72	11.08	6.26	4.55	3.45	2.77	1.94	5.62	4.80

### 2.3 Pre-treatment of the biomass ash

Biomass ash was sieved with 800 μm size sieve for the removal of unburnt carbon and coarse impurities. The particle size reduction of the ash was then performed with a planetary ball mill for 30 min with a rotational speed of 150 rpm. 25 g of fly ash was dissolved in 250 mL 4 M HCl solution in a glass reactor (liquid to solid (L/S) ratio of 10) at temperature 21 ± 1°С for 24 h under continuous mechanical mixing at 300 rpm. The Si-rich solid fraction was separated from the liquid phase after centrifugation at 3750 rpm for 10 min and filtration with a pore size of 8 μm, after which it was subsequently washed, dried in an oven at 105 °C and crushed in a mortar with a pestle.

### 2.4 Zeolite synthesis

NaP and LTA zeolites were synthesised by alkaline fusion-assisted hydrothermal process in three steps [11,12]: (1) alkali fusion, for the transformation of the crystalline phases into reactive amorphous phases, (2) ageing, for the dissolution of amorphous aluminosilicate species at room temperature under continuous mixing, (3) hydrothermal treatment, for the crystallisation of the dissolved species. Pre-treated biomass ash was mixed with NaOH pellets (and with NaAlO_2_ powder in the case of LTA synthesis, with ash/NaAlO_2_ mass ratio of 1.50), crushed, and fused inside a furnace for 3 h at 600 °C. Once the fusion was completed, the solids were ground, mixed with deionised water, and transferred to a Teflon-lined reactor for ageing at room temperature for 24 h. After ageing, the reactor was placed inside a stainless-steel vessel, and left in an oven at different temperatures and durations for the hydrothermal treatment. Finally, the sample was taken from the oven, filtered, thoroughly washed with deionised water until pH 8, dried at 105 °C, and ground. The experimental design demonstrating the parameters studied throughout the investigation is presented in Table 2.

**Table 2:** Experimental parameters used in the study for the investigation of zeolite synthesis.

Zeolite	NaOH/ash (wt./wt.)	Crystallisation temperature, °C	Crystallisation time, h
NaP1	1	120	24
NaP2	1.33	120	24
NaP3	1.67	120	24
NaP4	2	120	24
NaP5	2	90	24
NaP6	2	150	24
LTA1	0.67	90	24
LTA2	1	90	24
LTA3	1.33	90	24
LTA4	1.67	90	24
LTA5	1	60	24
LTA6	1	120	24
LTA7	1	90	3
LTA8	1	90	6
LTA9	1	90	12

### 2.5 Adsorption study

The preliminary adsorption performance of Cu(II), Zn(II), Cd(II) and Pb(II) using the NaP and LTA zeolites was investigated with batch adsorption experiments at temperature 24 ± 1 °C, with the adsorbate concentration, adsorbent dose, and contact time of 100 mg/L, 2.5 g/L, and 24 h, respectively. Adsorption properties of metals were calculated using Eq. (1) and Eq. (2) [1]:

(1) $$R=\frac{c_{0}-c_{t}}{c_{0}}\cdot 100\%\text{,}$$

(2) $$q_{t}=\frac{c_{0}-c_{t}}{m}\cdot V\text{,}$$

where R is the removal (%), $c_{0}$ and $c_{t}$ are metal concentrations in the beginning and different intervals of the reaction (mg/L), *V* is the solution volume (L), *m* is the adsorbent mass (g), $q_{t}$ is the adsorbed metal amount per g of adsorbent at different intervals (mg/g).

The effect of solution pH was investigated in multicomponent solutions at an adsorbent dosage of 2.5 g/L, reaction time of 24 h, and solution concentration of 100 mg/L. The effect of adsorbent dosage was investigated in multicomponent solutions at the initial solution pH (5.2), reaction time of 24 h, and solution concentration of 100 mg/L.

The equilibrium isotherm was identified with 10 mL of metal solution with a concentration range between 10 mg/L and 250 mg/L and between 50 mg/L and 500 mg/L for NaP and LTA zeolites, respectively. Isotherm studies were performed for Cu(II), Cd(II), and Pb(II) in their mono-component solutions. The studied adsorption isotherm models Langmuir, Freundlich, Temkin, Dubinin-Radushkevich, Sips and Redlich-Peterson are presented in Table S1.

Kinetic experiments were carried out for Cu(II), Zn(II), Cd(II) and Pb(II) in multicomponent solutions with concentrations of 100 and 150 mg/L for each metal. The adsorbent dose was 2.5 g/L of NaP and LTA at the initial solution pH at different contact times (5 min–6 h). The adsorption kinetics were analyzed with pseudo-first-order, pseudo-second-order, intra-particle diffusion and Boyd diffusion models are presented in Table S2.

All experiments were done in duplicate to ensure the statistical reliability of the results.

## 3 Results and discussion

### 3.1 Preliminary adsorption performance of NaP and LTA zeolites

**Fig. 1:**
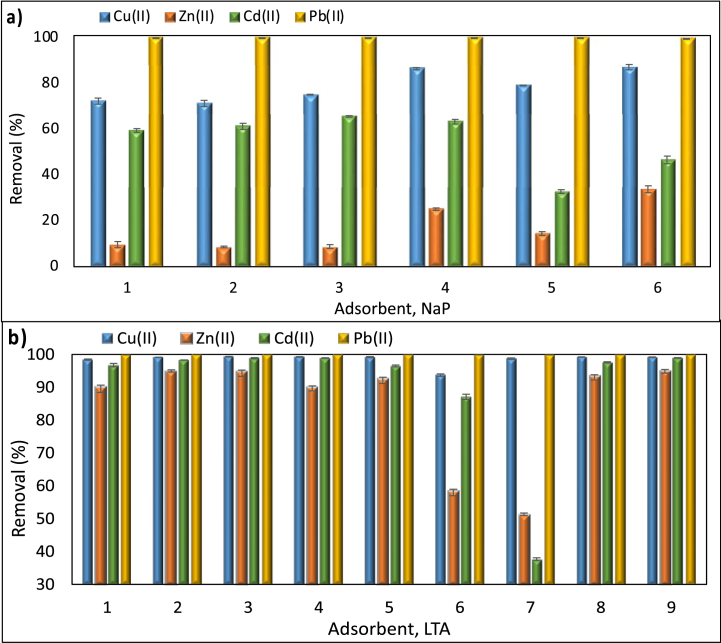
Simultaneous removal of metal ions with NaP (a) and LTA zeolites (b). Adsorbate concentration, adsorbent dose, pH, and contact time of 100 mg/L, 2.5 g/L, 5.2 and 24 h, respectively (Numbers shown in x axis refer to the synthesised materials that have been presented in Table 2).

Preliminary adsorption performance of the prepared zeolites for the hazardous metals Cu(II), Zn(II), Cd(II), and Pb(II) is presented in Fig. 1. Pb(II) removal efficiency was over 99% for all materials prepared, and both adsorbents showed a significant affinity for Pb(II) in the case of competitive adsorption. The removal efficiency had following tendency Pb(II) > Cu(II) > Cd(II) > Zn(II) for both NaP and LTA zeolites. This trend is attributed to the size of the hydrated ionic radii of Cu(II), Zn(II), Cd(II) and Pb(II) which are 0.295 nm, 0.430 nm, 0.426 nm and 0.261 nm [17,18]. More noticeable changes were observed between prepared zeolites for the adsorption of Cu(II), Cd(II) and Zn(II), which has also been supported in previous investigations [12,17].

In assessing the adsorption performance of NaP zeolites (Fig. 1a), the highest removal for Cu(II) and Cd(II) was achieved with NaP4, as 86.4% and 63.4%, respectively. The large differences in adsorption yields for Zn(II) and Cd(II) between NaP4, NaP5 and NaP6 products illustrate the vital influence of crystallisation temperature on performance of the prepared zeolites. Despite the incomplete crystallisation and highly amorphous structure observed, the adsorption performance of NaP1, NaP2 and NaP3 zeolite was also relatively high for Cd(II) and reached 59.4%, 61.2% and 65.6%, respectively. The adsorption performance of NaP5 (32.7%) and NaP6 (46.6%) for Cd(II) on the other hand was considerably lower when compared to that of NaP4. Hence, the crystallisation temperature had a greater effect on the removal of Zn(II) and Cd(II), while the removal of Cu(II) is affected by increasing the ratio between the NaOH and ash in the alkali fusion step.

As presented in Fig. 1b, crystallisation temperature of 90 °C, consistent with the previous studies [10,19] was sufficient to prepare highly adsorptive LTA zeolites. Thus, the relatively low removal efficiency for copper of LTA5 (99.0%) and LTA6 (93.7%) could be explained by low temperature of 60 °C that is insufficient for crystallisation, whereas the latter one contains the mixture of both LTA and sodalite due to the high temperature applied. The incomplete crystallisation in LTA7 after 3 h is confirmed with the XRD analysis, which will be presented in Fig. 6b, Section 3.2.3 and resulted in very poor adsorption performance for all three metals. As expected, the addition of NaAlO_2_ led to a substantial drop in the required NaOH amount, as the source of aluminium for the synthesis. The most important parameters to prepare LTA zeolites with the highest adsorption performance for Cu(II) 99.3%, Zn(II) 94.9% and Cd(II) 99.0% can be suggested to be the crystallisation temperature and time, followed by the NaOH/ash ratio. Along with the XRD and SEM data, the best zeolites for the adsorptive removal of Cu(II), Zn(II), Cd(II) and Pb(II) were chosen to be NaP4 and LTA9.

The results of BET analysis of the chosen adsorbents are presented in Table 3 and similar results have been reported for NaP [1,12,17,20] and LTA [10,21] in previous studies. The shape of the N_2_ adsorption-desorption isotherm of NaP zeolite with a hysteresis loop that can be seen in Fig. S2a indicate the characteristics of a mesoporous material. Additionally, the type of hysteresis indicates slit-shaped pores with non-rigid aggregates of plate-like particles [22]. Very low micropore volume of 0.0005 cm^3^/g and minor hysteresis of LTA zeolite (Fig. S2b) indicates the primarily macroporous nature of this material [22].

**Table 3:** BET characterisation results of NaP4 and LTA9 zeolite.

Materials	Surface area (m^2^/g)		Average adsorption pore diameter (nm)		Pore volume (cm^3^/g)	t-plot micropore volume (cm^3^/g)
	BET	BJH	BET	BJH		
NaP4	65.0	54.6	1.1	6.6	0.019	0.0074
LTA9	13.7	13.4	8.7	9.5	0.030	0.0005

#### 3.1.1 Influence of NaOH/ash ratio in alkali fusion stage

**Fig. 2:**
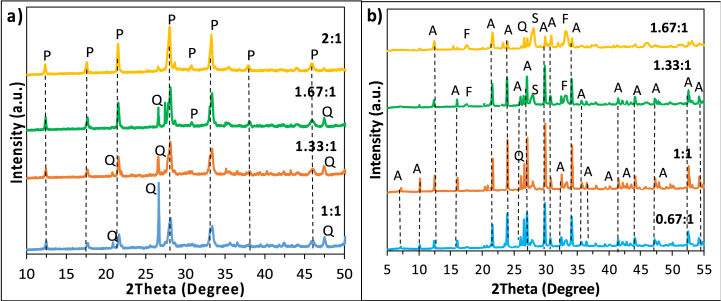
XRD diffractograms of NaP1-4 (a), LTA1-4 (b) showing the influence of NaOH/ash (wt./wt.) ratio on NaP and LTA zeolite. P: NaP zeolite, Q: quartz, A: LTA zeolite, F: faujasite, S: sodalite.

The XRD patterns of NaP and LTA zeolites are presented in Fig. 2.

According to Fig. 2a, the peaks appeared at 2θ = 12.3°, 17.5°, 21.5°, 28.0°, 33.2°, 37.9°, and 45.9° were assigned to NaP zeolite [20,23,24]; and LTA peaks appeared at 2θ = 7.3°, 10.2°, 12.5°, 16.1°, 21.7°, 24.0°, 26.1°, 27.1°, 30.0°, 30.8°, 32.5°, 33.4°, 34.2°, 35.7°, 36.5°, 44.2°, 47.3°, 47.9°, 52.6°, and 54.3° [23,25,26]. As can be seen from Fig. 2a, NaOH/ash ratio of 1, 1.33, and 1.67 did not provide sufficient NaOH for the complete reaction of silica in the form of quartz. The relative intensity of the main quartz peak (2θ = 26.7°) compared to characteristic NaP peaks decreases with increasing NaOH amount, and the peak disappears when the ratio is 2.0 [27]. Fig. 2b compares the patterns of LTA zeolites prepared at a NaOH/ash ratio of 0.67, 1, 1.33, and 1.67. The relative intensity of the major quartz peak against the characteristic LTA peaks was at its minimum value for NaOH/ash ratio of 1.

**Fig. 3:**
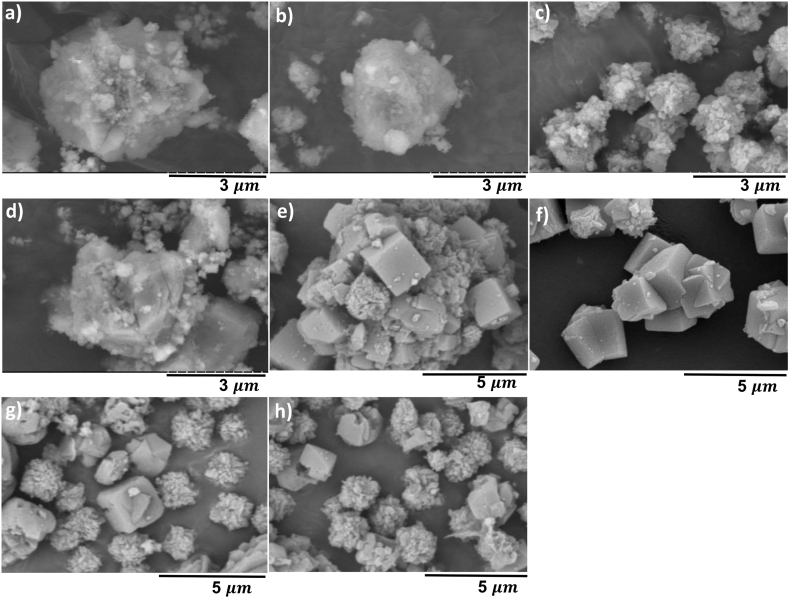
SEM images of NaP1-4 (a–d) and LTA1-4 (e–h) showing the influence of NaOH/ash (wt./wt.) ratio on NaP and LTA zeolite.

Fig. 3a–d presents SEM images of NaP1–NaP4. The small agglomerates composed of relatively elongated grains which can be noticed in Fig. 3d indicate the successful synthesis of NaP zeolite [28,29]. The amorphous fine material formed in Fig. 3e alongside the characteristic cubes of LTA zeolite might be due to the incomplete crystallisation proposing that the NaOH/ash ratio of 0.67 did not provide sufficient alkalinity under the applied condition, which finds support from the literature [30,31]. Furthermore, when the NaOH/ash ratio is 1.33 and 1.67 a sodalite peak appeared at 2θ = 28.1° (Fig. 2b), which can be also observed in Fig. 3g–h. This result may be explained by the fact that the excess NaOH dissolved during the hydrothermal treatment, and it formed sodalite through supersaturation which was previously reported in other studies [26,32,33]. Finally, the reduction in the particle size of the obtained LTA zeolites with increasing alkalinity can be explained by the accelerated nucleation rate and chemical reaction between silicates and aluminates [34].

#### 3.1.2 Influence of hydrothermal treatment temperature

**Fig. 4:**
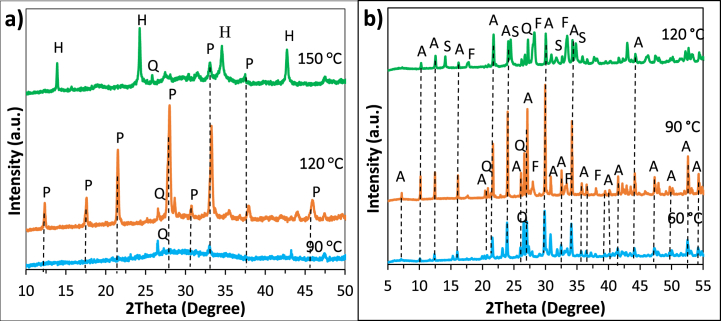
XRD diffractograms of NaP4-6 (a) and LTA4-6 (b) showing the influence of hydrothermal treatment temperature on NaP and LTA zeolite. P: NaP zeolite, H: hydroxysodalite, Q: quartz, A: LTA zeolite, F: faujasite, S: sodalite.

The influence of the crystallisation temperature on the crystal structure and the morphology of the products is presented in Fig. 4a. Strong relationship between the crystallisation temperature and zeolite structure has been reported in the literature [9,35].

**Fig. 5:**
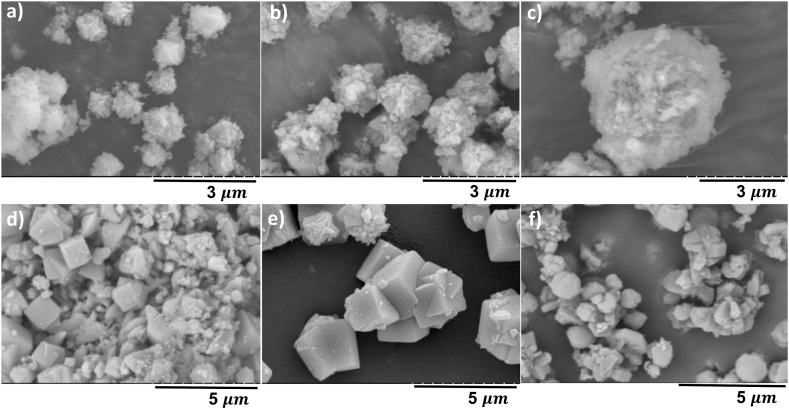
SEM images of NaP5 (a), NaP4 (b), NaP6 (c) and LTA5 (d), LTA6 (e) and LTA7 (f) showing the influence of hydrothermal treatment temperature on NaP and LTA zeolite.

Low crystallisation temperatures accelerate the dissolution rate of aluminosilicates and lead to a great number of crystal nuclei with smaller sizes [1]. The characteristic peaks of NaP zeolite (Fig. 4a) emerged first at 120 °C, which is confirmed with the rounded crystals of octahedral shape shown in Fig. 5b. Temperature of 90 °C was not sufficient for complete crystallisation of the synthesis gel [25,35], that can be deduced by observing the heterogeneous product in Fig. 5a which contains particles of different size and shape. Crystallisation temperature of 150 °C promoted the transformation of NaP crystals to thermodynamically more stable hydroxy sodalite [35]. The transformation of NaP to hydroxy sodalite at 150 °C can be seen in Fig. 5c where the rounded octahedron crystals of NaP zeolite with concave faces are replaced with a yarn-ball crystal. As can be seen from Fig. 4b, the relative intensity of characteristic LTA zeolite peaks to other peaks has increased significantly at 90 °C compared to 60 °C, indicating that the transformation of the amorphous gel did not occur at 60 °C. Additional peaks of sodalite (SOD) and FAU-zeolite appeared as impurities at 120 °C. This formation may be explained by Ostwald rule of stages, where the formation of a metastable structure is followed by its transformation into thermodynamically more stable structures. Zeolites are metastable materials, and their crystallisation involves gradual dissolution of one phase in addition to nucleation and growth of a more stable structure. FAU and SOD are more stable materials compared to LTA due to their denser lattice; hence, it is considered that their formation was favoured at 120 °C [10,19,36,37]. The formation of FAU and SOD at 120 °C is confirmed with the SEM images (Fig. 5f) where the cubic crystals of LTA are replaced with octahedral crystals and yarn-ball shaped structures, respectively.

**Fig. 6:**
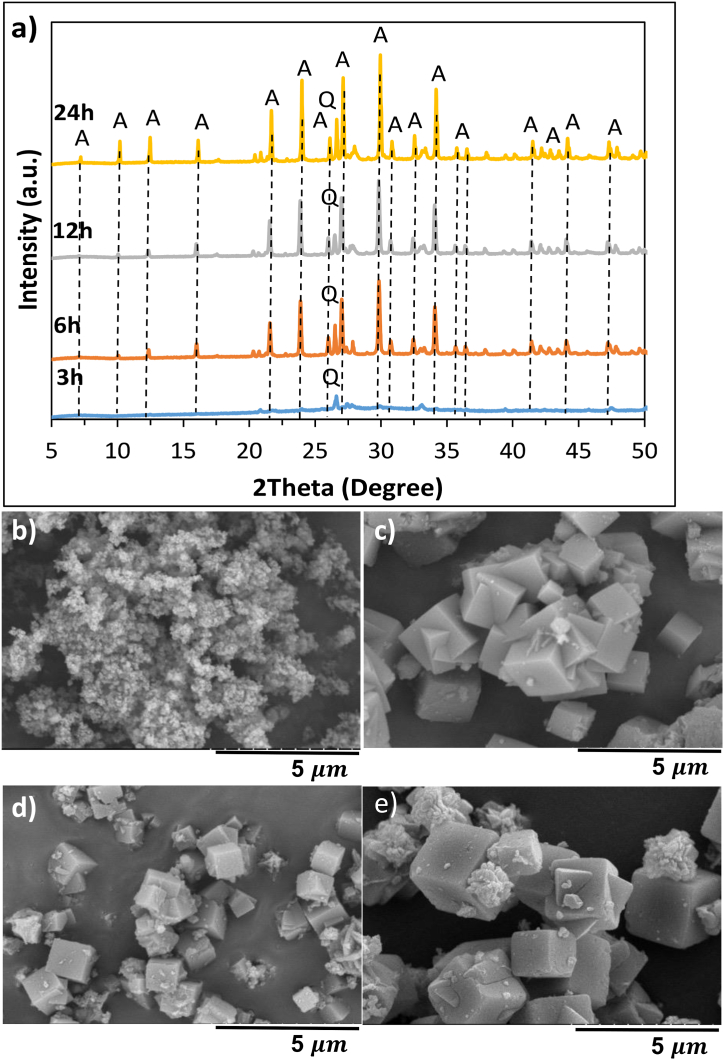
XRD diffractograms (a) and SEM images of LTA zeolite (b–e) showing the influence of hydrothermal treatment time on LTA zeolite. Hydrothermal treatment time of (b) 3 h, (c) 6 h, (d) 12 h, (e) 24 h. Q: quartz, A: LTA zeolite (NaOH/ash ratio of 1, hydrothermal treatment temperature: 90 °C).

#### 3.1.3 Influence of hydrothermal treatment time

The XRD patterns of LTA zeolites prepared under different hydrothermal treatment times (3, 6, 12 and 24 h) are shown in Fig. 6a. 3 h crystallisation time resulted in an amorphous structure showing no peaks, indicating that a higher reaction time is needed for the formation of LTA crystals. These results are consistent with those of Rozkhovskaya et al. [13], who showed that a crystalline LTA starts to form after 6 h. After 6 h of crystallisation time zeolite LTA was formed; however, the proportion of quartz peaks to the peaks of the LTA phase becomes minimum at 12 h of crystallisation time. The impact of the crystallisation time on the surface morphology of LTA is demonstrated in Fig. 6b–e. Although the characteristic cubical shape associated with LTA starts to form after 6 h, the surface defects can be noticed due to the large number of clustered particles. Further increase in crystallisation time after 12 h did not have an impact on the particle size of the synthesised zeolites. These findings accord with those of Jin et al. who investigated the effect of crystallisation time (2, 4, 6, 8, 10, 12 and 14 h) on the synthesis of LTA from coal gangue and reported the optimum time to be 10 h [10].

### 3.2 Systematic adsorption of Cu(II), Zn(II), Cd(II) and Pb(II) with chosen zeolites

#### 3.2.1 Influence of solution pH and adsorbent dosage

**Fig. 7:**
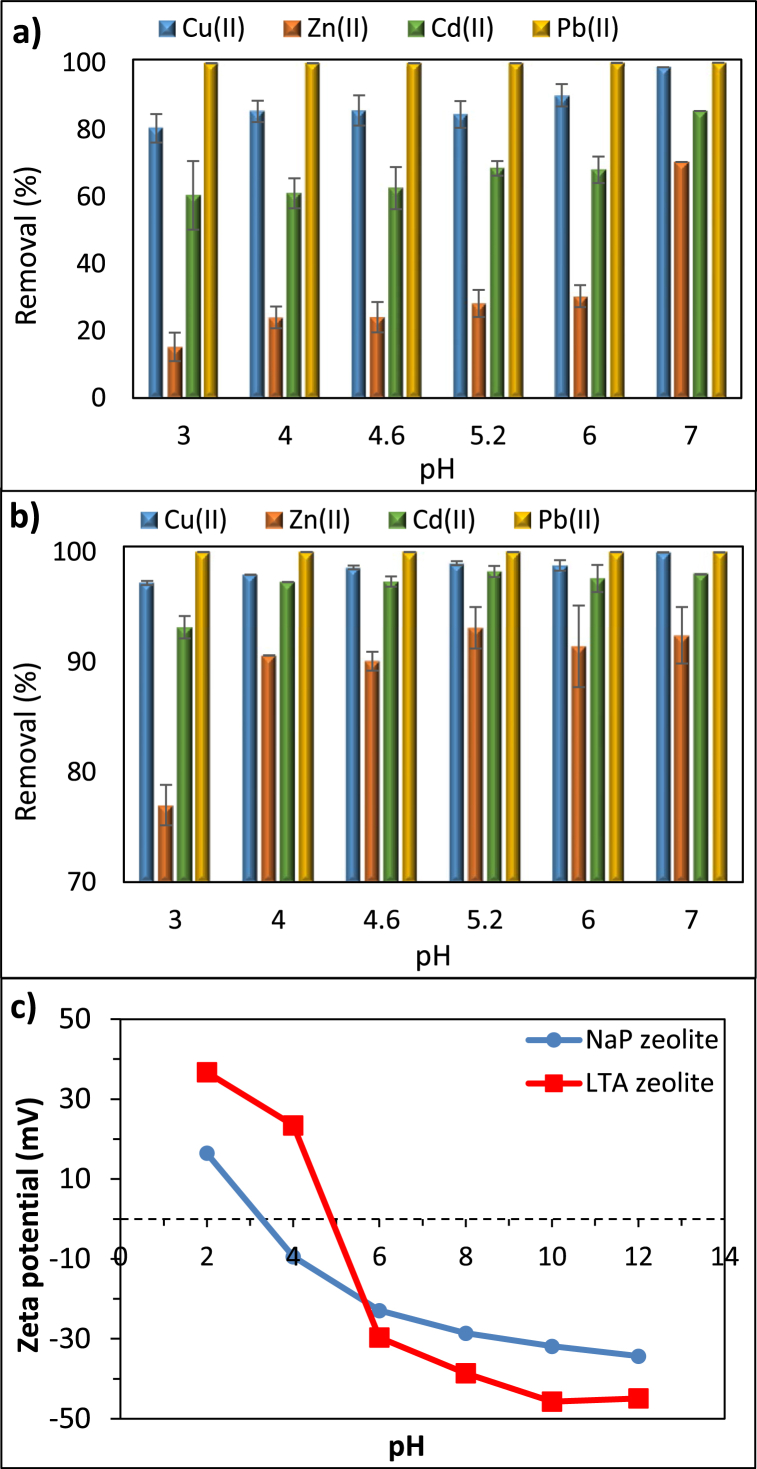
The effect of solution pH on the adsorption of metal ions on (a) NaP, (b) LTA zeolite, and (c) zeta potential analysis results. Adsorbate concentration, adsorbent dose, and contact time of 100 mg/L, 2.5 g/L, and 24 h, respectively.

Due to strong impact of solution pH on the surface characteristics of zeolite particles, it is essential to evaluate its effect into adsorption processes. The impact of pH on the metal removal and zeta potentials of the NaP and LTA zeolites are presented in Fig. 7.

According to Fig. 7a and b, pH increase enhanced the adsorption performance of both zeolites, whereas the zeta potential decreased from 16.5 to 36.7 mV to −34.3 and −45.7 mV with increased pH value for NaP and LTA zeolites, respectively. Adsorption performance of zeolites is low when the surface charge of the particles is high, which happens at strong acidic conditions. Metals are highly soluble in strong acidic conditions, thus, are in competition with H^+^ ions for the negatively charged adsorbent sites [1,26,38]. Furthermore, when the solution pH is lower than the isoelectric point of zeolites (pH < pH_IEP_), both the metal ions and the particles are positively charged and generate electrostatic repulsion which has a negative influence on the adsorption [1]. At high pH levels however, there is a greater attraction between negatively charged surface and metal cations as also illustrated in zeta potential measurement in Fig. 7c. The isoelectric points of NaP and LTA zeolites were measured as 3.2 and 5.0, respectively, indicating that at pH values above the pH_IEP_, the adsorption of positively charged metal ions on the zeolite surface may be enhanced [39]. Previous studies have reported similar results for NaP [40] and LTA zeolite [21].

Notwithstanding the high electrostatic repulsive forces, the adsorption performance of both adsorbents was high between pH 4 and pH7, except for the Zn(II) removal with NaP zeolite. Between pH 4 and pH 7, the removal efficiencies of Cu(II), Zn(II) and Cd(II) varied in the range of 85–100%, 24–70%, 60–80% for NaP zeolite. In the same pH range, the removal efficiencies of Cu(II), Zn(II) and Cd(II) were 98–99.5%, 90–92%, 97–99% for LTA zeolite. Pb(II) removal did not show any dependence on the solution pH and remained at 100% within this range. Nevertheless, at pH7, precipitation is the predominant removal mechanism, particularly in the case of Cu(II). Therefore, the adsorption mechanism of Cu(II), Zn(II), Cd(II) and Pb(II) could be explained as ion exchange, where these ions were replaced with the Na^+^ ions leaving the zeolite structure [26]. Due to the insignificant influence of pH on the adsorption of Zn(II), Cd(II) and Pb(II), and the small increase it resulted in Cu(II) adsorption between the pH of the initial solution (pH5.2) and pH6, the next steps of the study were performed at the unadjusted pH of the initial solution.

**Fig. 8:**
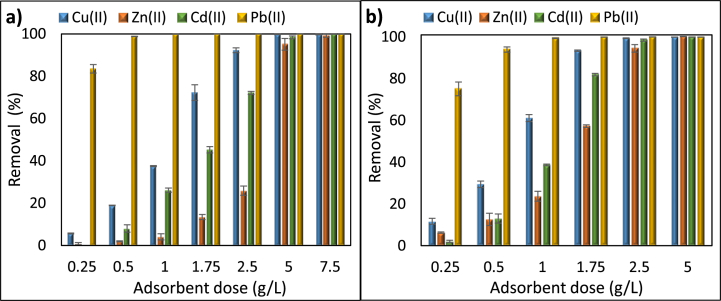
The effect of adsorbent dose on the adsorption of metal ions on (a) NaP and (b) LTA zeolite. Adsorbate concentration, solution pH, and contact time of 100 mg/L, 5.2, and 24 h, respectively.

To demonstrate the influence of the adsorbent dosage on resultant adsorptive properties, different loadings were applied at the optimum pH range chosen in the previous step, and the results are presented in Fig. 8. As shown in Fig. 8a and b, over 99% Pb(II) removal was achieved with a dose as low as 0.5 g/L with both NaP and LTA zeolites. More apparent changes were observed for Cu(II), Cd(II) and Zn(II). Complete adsorption of Cu(II), Cd(II) and Zn(II) took place with a NaP loading of 5, 5 and 7.5 g/L, respectively. In the case of LTA, the complete sorption of Cu(II) and Cd(II) was obtained by using a loading of 2.5 g/L, while the removal of Zn(II) was achieved with 5 g/L dosage. The unfavourable removal characteristics of the synthesised zeolites towards Zn(II) have been reported in previous studies [12], which may be due to the competition between metal ions for the active sites of the adsorbent and the greater size of hydrated Zn(II) ion, compared to that of Cu(II), Cd(II) and Pb(II) [17]. These findings are logical and consistent with a great deal of previous work [18,21], as more active sites were provided for adsorption by increasing the amount of adsorbent in the solution. As the current study focused on the low-cost aspect of the process in addition to its efficiency, next experiments were performed at an adsorbent dosage of 2.5 g/L.

#### 3.2.2 Adsorption isotherm

**Fig. 9:**
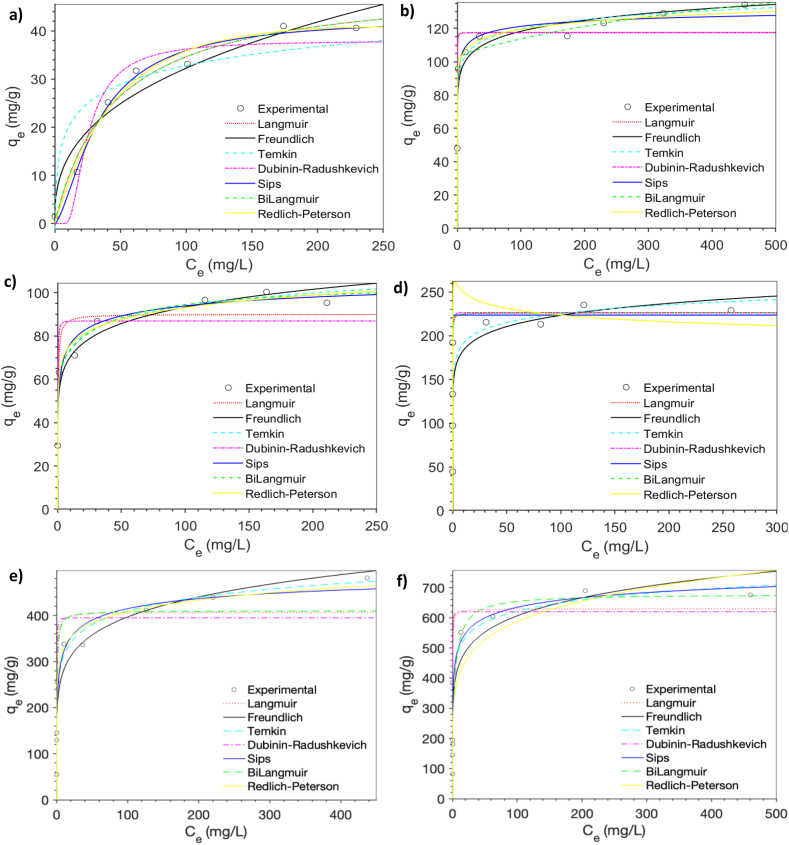
Adsorption isotherm model fittings for Cu(II) on (a) NaP and on (b) LTA zeolite, Cd(II) on (c) NaP and on (d) LTA zeolite, Pb(II) on (e) NaP and on (f) LTA zeolite. (Adsorbent dose: 2.5 g/L, reaction time: 24 h, pH: Initial pH of the solution, 5.2).

The adsorption performance of NaP and LTA zeolites for different initial metal concentrations are shown in Fig. 9a–f. The adsorption capacity of both zeolites increased until the equilibrium points with the concentration of metal ions, due to the increased driving force from concentration gradients. Consistent with the previous results (See Section 3.1), LTA zeolite exhibited a significantly greater adsorption capacity, compared to NaP zeolites. This may be explained by the greater crystallinity of LTA over NaP which was previously illustrated in Fig. 2, Fig. 4. Another possible reason for the high adsorption values is that NaP zeolite contains smaller pores which, according to the literature [14], may have impeded the sorption of metal ions. In fact, most of the hydrated metal ions could not enter the pores of NaP zeolite as their radii are bigger than that of NaP zeolite. These corresponding metals however may have gone through dehydration and entered the channels or pores of the zeolite materials and have been adsorbed via ion exchange, electrostatic attraction, or other manners of adsorption [1,41].

The hydration energy of metal ions plays a critical role in their dehydration characteristics. The metal ion having the lowest hydration energy is more likely to go through dehydration. The order of hydration energy of the corresponding metal ions is as following: Cu(II) > Cd(II) > Zn(II) > Pb(II) [41]. As Pb(II) has the lowest hydration energy compared to other metal ions, it can be said that its dehydration was more likely than that of other ions.

As can be noticed in Fig. 9, both NaP and LTA zeolites exhibited a higher adsorption capacity for Cu(II) than Cd(II). This is somewhat surprising regarding the results in multicomponent system, where the removal of Cu(II) was higher than that of Cd(II). Stronger affinity of zeolites in multicomponent solution for Cu(II) than for Cd(II) is attributed to the smaller size of the hydrated ionic radiuses of Cu(II), than that of Cd(II) [17,18].

As can be observed Fig. 9b, d and 9f, the steepness of the initial slope of the curves for LTA zeolite indicates a good affinity between metal ions and the sorbent [38]. For both zeolite materials, the Sips and Redlich-Peterson isotherm models provided the best fits for Cu(II), Cd(II), and Pb(II) ions. Sips model can be defined as a combination of Langmuir and Freundlich models and it indicates that physisorption (Freundlich) and chemisorption (Langmuir) mechanism are predominant at low and high concentrations, respectively. In the case of physisorption, there is a reversible adsorption process with weak van der Waals interactions between the adsorption pair [18]. Appropriate fit with Sips model suggests the presence of heterogeneous active sites on the adsorbent surface, which correlates with a previous study investigating the sorption of Cu(II), Cd(II) and Pb(II) with zeolite adsorbents [38]. These results are also supported by the Redlich-Peterson model fit results, where the heterogeneity factor n_RP_ ≠1 (see Tables S2 and S3), indicating a non-ideal heterogeneous adsorption. Redlich-Peterson isotherm model is an empirical equation, and it can be applied for either homogeneous or heterogeneous systems. If the Redlich-Peterson exponential constant n_RP_ was equal to 1, it would reduce to Langmuir isotherm model and indicate a homogeneous adsorption [42,43]. Table 4 provides a detailed note on the removal performance for Cd(II), Cu(II), and Pb(II) ions with different adsorbents.

**Table 4:** A review of hazardous metal adsorption performance of different adsorbents in synthetic wastewaters.

Targeted metal	Sorption capacity (mg/g)	Adsorbent	Concentration	Solution pH	Reference
Cd(II)	188.7	ZIF-L/GO	10–200 mg/L	6	[44]
Cd(II)	45.66	Magnetic biochar	50 mg/L	7.0	[45]
Cd(II)	35.5	LaFe@CS	0.2–5 mg/L	6.5	[46]
Cd(II)	265	Ag–Fe-MOF	50–200 mg/L	7	[47]
Cu(II)	84.65	Zeolite Na–P1	40–200 mg/L	3	[48]
Cu(II)	110.5	DTPA-chitosan/alginate composite beads	20–500 mg/L	3	[49]
Cu(II)	32.0	Carbon nanotube sheets	5–35 mmol/L	4.2	[50]
Pb(II)	411.8	Covalent organic framework (Ni0.6Fe_2_.4O_4_-HT-COF)	100–700 mg/L	5	[51]
Pb(II)	431.6	Coal gangue derived NaY zeolite	10–200 mg/L	7	[52]
Cd(II) Cu(II)	44.64 33.76	Synthetic clinoptilolite	10–600 mg/L	5	[53]
Cd(II) Cu(II) Pb(II)	74.1 57.8 109.9	FAU zeolite	100–500 mg/L	–	[18]
Cd(II) Cu(II) Pb(II)	75.5 37.1 –	Methacrylate-Na-Y-Zeolite	5–100 mg/L	4.5	[38]
Cd(II) Cu(II) Pb(II)	26.9 20.9 88.3	NaP1 zeolite	0–295 mg/L 0–223.5 mg/L	5.6	[17]
Pb(II)	1385	CS/Fe-HAP composite bead	100–700 mg/L		[54]
Pb(II)	497.01	Zeolite P	10–1000 mg/L	5.0	[55]
Cu(II)	175	Cellulose-g- Poly(acrylamide)	1–3.6 mol/L	5.8	[56]
Cd(II) Cu(II) Pb(II)	117.3 42.9 534.3	NaP zeolite	10–250 mg/L	5.2	This study
Cd(II) Cu(II) Pb(II)	223.5 140.1 850.7	LTA zeolite	50–500 mg/L		

#### 3.2.3 Adsorption kinetics

**Fig. 10:**
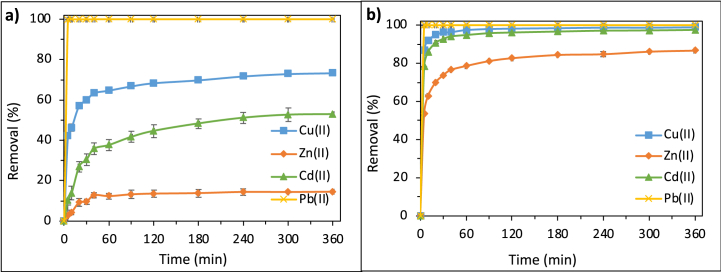
Effect of adsorption time on the adsorption performance of (a) NaP and (b) LTA zeolite in 100 ppm solution (Adsorbent dose: 2.5 g/L, pH: Initial pH of the solution, 5.2).

Sorption curves as a function of time for NaP and LTA zeolites are shown in Fig. 10a and b. The adsorption of Cu(II), Zn(II), and Cd(II) was obtained in 360 min, while over 80% of the overall removal was achieved in 180 min. Pb(II) adsorption however, reached its maximum value (100%) within 5 min independent of the adsorbent material or the solution concentration. The significantly faster adsorption rate for Pb(II) accords with those of other studies [17,18]. This superiority can be explained by lower hydrolysis constant and higher electronegativity value of Pb(II) compared to other metals (Table 5) which favours the formation of Pb_2_(OH)^3+^ and Pb_3_(OH)^4+^ whose sorption is much higher and faster than other metal cations [17].

**Table 5:** Hydrolysis constants and electronegativity values of the studied metals [17].

Metal cation	Cu(II)	Zn(II)	Cd(II)	Pb(II)
Hydrolysis constant (logKi)	8.0	8.96	10.80	7.71
Pauling electronegativity	1.95	1.65	1.69	2.33

The kinetic data were analyzed with 4 kinetic models: pseudo-first-order, pseudo-second-order, intraparticle diffusion (IPD) and Boyd models, and the results of the best fitting models are presented in Table 6. Parameters of IPD and model fitting results can be found in Table S5 and Figs. S7 and S8, respectively. The rate constants *k*_*1*_ and *k*_*2*_ indicate how quickly the adsorption occurs and are both proportional to the reaction speed. According to the correlation coefficient *R*^*2*^ and accurate estimation of *q*_*e*_ with _qm_, the pseudo-second-order model achieved the best fit for all metals which also accords with previous studies [1,17,18]. The only exception is that of Zn(II) adsorption with NaP zeolite at an initial concentration of 150 ppm. Low correlation factor obtained for all kinetic models could be attributed to the poor Zn(II) removal performance and low adsorption capacity, which was also observed in the isotherm study. Furthermore, all correlation factors calculated for Pb(II) adsorption had the value 1 due to exceptional sorption performance of both NaP and LTA towards this metal. In general, pseudo-first-order kinetic model provides good fits for a large range of contact time and it can be applied during the initial stages of adsorption [57]. Additionally, it was previously presented that pseudo-second-order model gives better fit than the pseudo-first-order model when the initial adsorbate concentration is relatively low [58]. Pseudo-second-order kinetic model assumes that chemisorption interaction might be the rate-limiting step [17,59].

**Table 6:** Pseudo-first-order and pseudo-second-order kinetics results of NaP and LTA zeolites for 100 and 150 mg/L metal solutions.

Zeolite	Model	Parameters	100 mg/L				150 mg/L			
			Cu	Zn	Cd	Pb	Cu	Zn	Cd	Pb
NaP		q_exp_ (mg/g)	30.52	5.94	23.52	37.44	32.76	2.00	23.56	56.02
	PFO	q_m,_ (mg/g)	26.58	5.07	19.81	37.44	27.51	2.85	18.61	56.02
		k_1_ (1/min)	0.144	0.037	0.031	1.363	0.201	0.330	0.076	1.314
		R^2^	0.925	0.967	0.943	1	0.923	0.523	0.885	1
	PSO	q_m,_ (mg/g)	28.01	5.60	21.99	37.08	28.77	2.80	20.08	56.79
		k_2_ (g/[mg·min])	0.008	0.009	0.002	7.186	0.011	5.621	0.005	2.398
		R^2^	0.978	0.975	0.985	1	0.967	0.492	0.953	1
LTA		q_e_ (mg/g)	37.58	33.96	36.79	37.07	50.25	18.52	41.30	56.01
	PFO	q_m,_ (mg/g)	36.93	30.60	35.62	37.08	46.09	17.42	38.00	55.99
		k_1_ (1/min)	0.422	0.167	0.318	1.594	0.273	0.329	0.272	1.363
		R^2^	0.996	0.945	0.990	1	0.957	0.953	0.961	1
	PSO	q_m_ (mg/g)	37.42	32.08	36.29	37.08	47.60	17.84	39.21	56.00
		k_2_ (g/[mg·min])	0.037	0.008	0.021	13.49	0.011	0.042	0.008	2.576
		R^2^	0.999	0.988	0.999	1	0.986	0.971	0.988	1

Both pseudo-first- and pseudo-second-order kinetic models assume that the adsorption kinetics is controlled by the surface reaction. The intra-particle diffusion and Boyd models were also studied to identify if the rate-controlling step of the adsorption process is particle diffusion or film diffusion, where the transport of the metals occurred within the pores or to the external surface of the adsorbent, respectively [60]. The linear fitting of the experimental data did not pass through the origin in either Intra-particle diffusion model or Boyd model (Figs. S7 and S10). This can be interpreted that both diffusion mechanisms took place simultaneously, but film diffusion is the main rate-controlling step in the adsorption process. Furthermore, the multi-linearity observed in the intra-particle diffusion model plot of adsorption capacity vs. square root of time shows that the adsorption process is formed via different stages in the case of NaP zeolite. These linear portions might be corresponding to the external surface adsorption stage (diffusion in macropores), gradual adsorption stage (intra-particle diffusion in mesopores), and equilibrium stage (diffusion in micropores), respectively [42,61,62].

## 4 Conclusions

NaP and LTA zeolites were successfully synthesised using woody biomass ash as the raw material by the fusion-assisted hydrothermal method after the acid dissolution of the ash for impurity removal. NaAlO_2_ was utilized to adjust the required Si/Al ratio for LTA zeolite, while no additional aluminium or silica source was used for NaP synthesis, which is a critical aspect for reducing the synthesis costs. The optimum synthesis conditions were determined according to the crystal structure, morphology and sorption performance of the synthesised zeolites for metals Cu(II), Zn(II), Cd(II) and Pb(II). The optimum NaOH/ash ratio and crystallisation temperature were: 2 and 120 °C for NaP zeolite; and 1 and 90 °C for LTA zeolite, while the optimum crystallisation time was 12 h for LTA zeolite. Synthesised NaP and LTA zeolites under the optimum conditions exhibited a specific surface area of 65.03 m^2^/g and 13.69 m^2^/g, respectively. Sips and Redlich-Peterson models were the best to describe the adsorption, indicating a non-ideal heterogeneous adsorption. Pseudo-second order kinetic matched well with the adsorption of all metal ions indicating that chemisorption interaction might be the rate-controlling step in the adsorption process. Maximum adsorption capacity of NaP zeolite was 42.9 mg/g for Cu(II) and 117.3 mg/g for Cd(II), while those of LTA zeolite were 140.1 mg/g and 223.5 mg/g for LTA zeolite, respectively. The affinity of NaP and LTA zeolite had the trend Pb(II) > Cu(II) > Cd(II) > Zn(II) in multicomponent solutions. This study has shown that despite their high impurity content, biomass ash is a promising low-cost raw material for the synthesis of both NaP and LTA zeolites with excellent sorption capacity for metal ions, and the standard zeolite synthesis process can be tailored for biomass ashes. A further study could assess the comparison of these zeolites with commercial NaP and LTA and investigate their adsorption performance for other pollutants.

## Author contribution statement

Mehmet Emin Küçük, M.Sc: Conceived and designed the experiments; Performed the experiments; Analyzed and interpreted the data; Contributed reagents, materials, analysis tools or data; Wrote the paper.

Iryna Makarava, Doctor of Science; Teemu Kinnarinen, Doctor of Science: Analyzed and interpreted the data; Contributed reagents, materials, analysis tools or data.

Antti Häkkinen, Doctor of Science: Contributed reagents, materials, analysis tools or data.

## Data availability statement

Data will be made available on request.

## Declaration of competing interest

The authors declare that they have no known competing financial interests or personal relationships that could have appeared to influence the work reported in this paper.
