# Supplementary material for: Simultaneous adsorption of Cu(II), Zn(II), Cd(II) and Pb(II) from synthetic wastewater using NaP and LTA zeolites prepared from biomass fly ash
Source: Heliyon. 2023 Sep 21;9(10):e20253. doi: 10.1016/j.heliyon.2023.e20253 (PMC10556607; doi:10.1016/j.heliyon.2023.e20253)
Supplement: Multimedia component 1 [file mmc1.docx]

**Simultaneous adsorption of Cu(II), Zn(II), Cd(II) and Pb(II) from synthetic wastewater using NaP and LTA zeolites prepared from biomass fly ash**

Mehmet Emin Küçük*, Iryna Makarava, Teemu Kinnarinen, Antti Häkkinen

Department of Separation Science, LUT School of Engineering Science, Lappeenranta-Lahti University of Technology LUT, Yliopistonkatu 34, FI-53850 Lappeenranta, Finland

*Corresponding author: E-mail address: mehmet.kucuk@student.lut.fi

**SUPPLEMENTARY DATA**

**S1. Adsorption study**

The preliminary adsorption performance of Cu(II), Zn(II), Cd(II) and Pb(II) using the NaP and LTA zeolites was investigated with batch adsorption experiments at temperature 24±1 °C, with the adsorbate concentration, adsorbent dose, and contact time of 100 mg/L, 2.5 g/L, and 24 h, respectively. Adsorption properties of metals were calculated using Eq. (1) and Eq. (2) [1]:

$R=\frac{c_{0}-c_{t}}{c_{0}}\cdot100\%$ (1)

$q_{t}=\frac{c_{0}-c_{t}}{m}\cdot V$ (2)

Where $R$ is the removal (%),$c_{0}$and$c_{t}$are metal concentrations in the beginning and different intervals of the reaction (mg/L), *V* is the solution volume (L), *m* is the adsorbent mass (g),$q_{t}$is the adsorbed metal amount per g of adsorbent at different intervals (mg/g).

The equilibrium isotherm was identified with 10 mL of metal solution with a concentration range between 10 mg/L and 250 mg/L and between 50 mg/L and 500 mg/L for NaP and LTA zeolites, respectively. Isotherm studies were performed for Cu(II), Cd(II), and Pb(II) in their mono-component solutions. The studied adsorption isotherm models Langmuir, Freundlich, Temkin, Dubinin-Radushkevich, Sips and Redlich-Peterson are presented in Table S1 [2-4].

**Table S1**

Isotherm models applied in this study.

| Model | Equation | Reference |
| --- | --- | --- |
| Langmuir | $q_{e}= \frac{{C_{e}K_{L}q}_{m}}{1+C_{e}K_{L}}$ | [2] |
| Freundlich | $q_{e}= C_{e}^{1/n}K_{F}$ | [3] |
| Temkin | $q_{e}= B_{Te}*log(a_{Te}C_{e})$ | [3] |
| Dubinin-Radushkevich | $q_{e}=Q_{D}*exp(-b_{D}*\left( \log\left( 1+\frac{1}{C_{e}} \right) \right)^{2})$ | [4] |
| Sips | $q_{e}= \frac{{{q_{m}{(K}_{S}C}_{e})}^{n_{S}}}{1+{{{(K}_{S}C}_{e})}^{n_{S}}}$ | [3] |
| Redlich-Peterson | $q_{e}= \frac{{q_{m}{(K}_{RP}C}_{e})}{1+{{{(K}_{RP}C}_{e})}^{n_{RP}}}$ | [3] |

Parameters: $C_{e}$: Metal concentration at equilibrium (mg/L), $q_{e}$: adsorbed metal amount at equilibrium (mg/g), $q_{m}$: adsorption capacity of the sorbent (mg/g). *K_L_*: Langmuir affinity constant (L/mg), *K_F_*: Freundlich affinity constant (L/mg), *1/n*: dimensionless Freundlich adsorption constant, *B_Te_*: Temkin constant related to the heat of adsorption (J/mg), *a_Te_*: equilibrium binding constant (L/mg), *Q_D_*: Dubinin-Radushkevich adsorption capacity (mg/g), *b_D_*: Activity coefficient related to mean adsorption energy (mol^2^/kJ^2^), *K_S_*: Sips affinity constant (L/mg), *n_S_*: Sips heterogeneity factor, *K_RP_*: Redlich-Peterson affinity constant (L/mg), *n_RP_*: Redlich-Peterson heterogeneity factor.

Kinetic experiments were carried out for metal solutions with concentrations of 100 and 150 mg/L. The adsorbent dose was 2.5 g/L of NaP and LTA at different contact times (5 min – 6 h). The adsorption kinetics were analysed with pseudo-first-order, pseudo-second-order, intra-particle diffusion and Boyd diffusion models are presented in Table S2 [1-2, 5].

**Table S2**

Kinetic models applied in this study.

| Model | Equation | Reference |
| --- | --- | --- |
| Pseudo-first order model | $q_{t}= q_{e}(1-e^{-k1t})$ | [1] |
| Pseudo-second order model | $q_{t}=\frac{k_{2}q_{e}^{2}t}{1+k_{2}q_{e}t}$ | [1] |
| Intra-particle diffusion model | $q=k_{dif}t^{0.5}(+C)$ | [2] |
| Boyd diffusion model | $B_{t}=-0.497-\ln\left( 1-F \right)$  $F=\frac{q_{t}}{q_{e}}$  $B_{t}=\frac{D_{i}\pi^{2}}{r^{2}}$ | [5] |

Parameters: $q_{t}$: adsorption capacity at time t (mg/g), $q_{e}$: adsorption capacity at equilibrium, Pseudo-first order model: *k_1_*: rate constant (min^-1^), Pseudo-second order model: $k_{2}$ rate constant (g/[mg.min]). Intra-particle diffusion model: *k_dif_*: diffusion rate constant (mg/[g .min^0.5^]) and *C*: the thickness of the boundary layer (mg/g). Boyd diffusion model: *F*: the fraction of metal ion adsorbed at any time t, *D_i_:* effective diffusion coefficient (cm^2^/s); *r:* radius of the adsorbent particle assuming spherical shape (cm).

All experiments were done in duplicate to ensure statistical reliability of the results.

**S2. FTIR analysis**

The FTIR spectra of the samples are presented in Fig. S1a, and the magnified range of 900-400 cm^-1^ is presented in Fig. S1b. The broad bands centring at 3374 cm^-1^ and 1633 cm^-1^ in the case of the leaching product were attributed to the presence of -OH stretching and bending vibrations of adsorbed water, respectively [6]. In the case of raw ash, the -OH functional group does not exist, indicating the absence of moisture. The peaks observed for raw ash at 1411 cm^-1^ and 875 cm^-1^ were associated with asymmetric stretching and asymmetric tensile vibrations of carbonate [6-7]. The peak that appeared at 677 cm^-1^ was associated with CaSO_4_ in the form of anhydrite, which was reported in the XRD analysis [7]. The peaks corresponding to carbonate and anhydrite disappeared in the leaching product, indicating that these minerals dissolved in the acid solution. Two peaks observed at 594 and 612 cm^-1^ in the raw ash were attributed to symmetric stretching vibrations of TO_4_ (T = Si and Al) [1]. Asymmetric stretching vibrations of Si-O appeared at around 964-1110 cm^-1^ for all 4 samples [7-9]. The broad bands centring at 3371-3391 cm^-1^ and 1642-1646 cm^-1^ for NaP and LTA zeolite were attributed to -OH stretching and bending vibration mode of the silanol groups due to zeolite hydration [10-11]. The peaks observed in the range of 500-1000 cm^-1^ are the skeletal vibration bands of NaP zeolite [12]. The reported peaks at 742 and 592 cm^-1^ were associated with the symmetric stretch vibrational peak of Si-O or Al-O [8]. The peaks at 976 cm^-1^ and 666 cm^-1^ in LTA zeolite were attributed to Si-O-Al symmetric and asymmetric stretching vibrations, respectively [8, 13]. The peak appeared at 548 cm^-1^ is considered as a fingerprint of LTA zeolite and it indicates the presence of a double ring (D4R) connection to sodalite cages [13]. The bending vibration of tetrahedron (TO4) was observed at around 431-460 cm^-1^ for the leaching product, NaP zeolite and LTA zeolite [8, 13-14].

**Fig. S1.** Absorbance infrared spectra of samples, GA: Ground ash, LA: Ash after acid leaching, NaP: NaP4 zeolite, LTA: LTA9 zeolite.

**S3. N_2_ adsorption-desorption isotherms**

Nitrogen adsorption-desorption isotherms at -196 C of (a) NaP and (b) LTA zeolites are demonstrated in Fig. S2.

**Fig. S2.** Nitrogen adsorption-desorption isotherms at -196 C of (a) NaP4 zeolite and (b) LTA9 zeolite.

**S4. Adsorption isotherm**

Isotherm modelling results for NaP and LTA zeolite are presented in Table S2 and Table S3.

**Table S2**

Adsorption isotherm model fittings for Cu(II), Cd(II), and Pb(II) on NaP zeolite.

| Cu(II) |  | |  |  |  |  |  |  |  |  |  |  |
| --- | --- | --- | --- | --- | --- | --- | --- | --- | --- | --- | --- | --- |
| q_e_ (mg/g) | | 41.04 |  |  |  |  |  |  |  |  |  |  |
| Langmuir | | | Freundlich | | Temkin | | Dubinin-Rad | | Sips | | Redlich-Pet. | |
| R^2^ | 0.981 | | R^2^ | 0.942 | R^2^ | 0.846 | R^2^ | 0.958 | R^2^ | 0.989 | R^2^ | 0.986 |
| Adj-R^2^ | 0.977 | | Adj-R^2^ | 0.931 | Adj-R^2^ | 0.815 | Adj-R^2^ | 0.949 | Adj-R^2^ | 0.984 | Adj-R^2^ | 0.979 |
| K_L_ | 0.0225 | | K_F_ | 5.727 | B_Te_ | 5.677 | Q_D_ | 38.03 | K_S_ | 0.030 | K_RP_ | 0.005 |
| q_m_ | 50.04 | | n_F_ | 2.664 | a_Te_ | 3.236 | b_D_ | 488.6 | n_S_ | 1.499 | n_RP_ | 1.212 |
|  |  | |  |  |  |  |  |  | q_m_ | 42.93 | q_mRP_ | 162.6 |
| Cd(II) |  | |  |  |  |  |  |  |  |  |  |  |
| q_e_ (mg/g) | | 100.4 |  |  |  |  |  |  |  |  |  |  |
| Langmuir | | | Freundlich | | Temkin | | Dubinin-Rad | | Sips | | Redlich-Pet. | |
| R^2^ | 0.835 | | R^2^ | 0.929 | R^2^ | 0.862 | R^2^ | 0.759 | R^2^ | 0.961 | R^2^ | 0.975 |
| Adj-R^2^ | 0.802 | | Adj-R^2^ | 0.915 | Adj-R^2^ | 0.955 | Adj-R^2^ | 0.711 | Adj-R^2^ | 0.943 | Adj-R^2^ | 0.962 |
| K_L_ | 3.08 | | K_F_ | 53.14 | B_Te_ | 8.628 | Q_D_ | 86.95 | K_S_ | 0.641 | K_RP_ | 12.84 |
| q_m_ | 90.05 | | n_F_ | 8.181 | a_Te_ | 529.3 | b_D_ | 0.194 | n_S_ | 0.334 | n_RP_ | 0.914 |
|  |  | |  |  |  |  |  |  | q_m_ | 117.3 | q_mRP_ | 62.9 |
| Pb(II) |  | |  |  |  |  |  |  |  |  |  |  |
| Qe (mg/g) 481 | | |  |  |  |  |  |  |  |  |  |  |
| Langmuir | | | Freundlich | | Temkin | | Dubinin-Rad | | Sips | | Redlich-Pet. | |
| R^2^ | 0.890 | | R^2^ | 0.906 | R^2^ | 0.937 | R^2^ | 0.859 | R^2^ | 0.930 | R^2^ | 0.951 |
| Adj-R^2^ | 0.875 | | Adj-R^2^ | 0.892 | Adj-R^2^ | 0.928 | Adj-R^2^ | 0.839 | Adj-R^2^ | 0.907 | Adj-R^2^ | 0.934 |
| K_L_ | 2.51 | | K_F_ | 198.7 | B_Te_ | 42.99 | Q_D_ | 394.9 | K_S_ | 0.308 | K_RP_ | 6.36 |
| q_m_ | 406.7 | | n_F_ | 6.66 | a_Te_ | 138.3 | b_D_ | 0.308 | n_S_ | 0.363 | n_RP_ | 0.914 |
|  |  | |  |  |  |  |  |  | q_m_ | 534.3 | q_mRP_ | 275.8 |

**Table S3**

Adsorption isotherm model fittings for Cu(II), Cd(II) and Pb(II) on LTA zeolite.

| Cu(II) |  | | | |  |  |  |  |  |  |  |  |  |  |
| --- | --- | --- | --- | --- | --- | --- | --- | --- | --- | --- | --- | --- | --- | --- |
| q_e_ (mg/g) | | 134.2 | | |  |  |  |  |  |  |  |  |  |  |
| Langmuir | | | | Freundlich | | | Temkin | | Dubinin-Rad | | Sips | | Redlich-Pet. | |
| R^2^ | 0.821 | | | R^2^ | | 0.913 | R^2^ | 0.954 | R^2^ | 0.813 | R^2^ | 0.968 | R^2^ | 0.982 |
| Adj-R^2^ | 0.791 | | | Adj-R^2^ | | 0.899 | Adj-R^2^ | 0.946 | Adj-R^2^ | 0.782 | Adj-R^2^ | 0.956 | Adj-R^2^ | 0.974 |
| K_L_ | 24.94 | | | K_F_ | | 81.96 | B_Te_ | 7.917 | Q_D_ | 117.4 | K_S_ | 5.215 | K_RP_ | 53.25 |
| q_m_ | 117.7 | | | n_F_ | | 12.56 | a_Te_ | 37100 | b_D_ | 0.067 | n_S_ | 0.297 | n_RP_ | 0.947 |
|  |  | | |  | |  |  |  |  |  | q_m_ | 140.1 | q_mRP_ | 93.66 |
| Cd(II) |  | | |  | |  |  |  |  |  |  |  |  |  |
| q_e_ (mg/g) | | | 235.2 | |  |  |  |  |  |  |  |  |  |  |
| Langmuir | | | | Freundlich | | | Temkin | | Dubinin-Rad | | Sips | | Redlich-Pet. | |
| R^2^ | 0.807 | | | R^2^ | | 0.683 | R^2^ | 0.702 | R^2^ | 0.794 | R^2^ | 0.957 | R^2^ | 0.823 |
| Adj-R^2^ | 0.775 | | | Adj-R^2^ | | 0.630 | Adj-R^2^ | 0.652 | Adj-R^2^ | 0.759 | Adj-R^2^ | 0.939 | Adj-R^2^ | 0.753 |
| K_L_ | 18.56 | | | K_F_ | | 150.3 | B_Te_ | 14.69 | Q_D_ | 226.4 | K_S_ | 18.06 | K_RP_ | 11.41 |
| q_m_ | 225.9 | | | n_F_ | | 11.64 | a_Te_ | 45230 | b_D_ | 0.0802 | n_S_ | 3.702 | n_RP_ | 1.051 |
|  |  | | |  | |  |  |  |  |  | q_m_ | 223.5 | q_mRP_ | 283 |
| Pb(II) |  | | |  | |  |  |  |  |  |  |  |  |  |
| q_e_ (mg/g) 689.1 | | | |  | |  |  |  |  |  |  |  |  |  |
| Langmuir | | | | Freundlich | | | Temkin | | Dubinin-Rad | | Sips | | Redlich-Pet | |
| R^2^ | 0.800 | | | R^2^ | | 0.863 | R^2^ | 0.873 | R^2^ | 0.770 | R^2^ | 0.889 | R^2^ | 0.843 |
| Adj-R^2^ | 0.777 | | | Adj-R^2^ | | 0.843 | Adj-R^2^ | 0.855 | Adj-R^2^ | 0.737 | Adj-R^2^ | 0.855 | Adj-R^2^ | 0.790 |
| K_L_ | 5.125 | | | K_F_ | | 352.2 | B_Te_ | 54.06 | Q_D_ | 620.2 | K_S_ | 0.361 | K_RP_ | 78890 |
| q_m_ | 630.2 | | | n_F_ | | 7.387 | a_Te_ | 986.2 | b_D_ | 0.177 | n_S_ | 0.300 | n_RP_ | 0.843 |
|  |  | | |  | |  |  |  |  |  | q_m_ | 850.7 | q_mRP_ | 285 |

**S5. Adsorption kinetics**

Kinetic modelling results for NaP and LTA zeolite are presented in Figs. S3-S6.

**Fig. S3.** Loading of (a) Cu(II), (b) Zn(II), (c) Cd(II) and (d) Pb(II) on NaP zeolite with time (Initial solution concentration: 100 mg/L, adsorbent dose: 2.5 g/L, pH: Initial pH of the solution, 5.2).

**Fig. S4.** Loading of (a) Cu(II), (b) Zn(II), (c) Cd(II) and (d) Pb(II) on NaP zeolite with time (Initial solution concentration: 150 mg/L, adsorbent dose: 2.5 g/L, pH: Initial pH of the solution, 5.2).

**Fig. S5.** Loading of (a) Cu(II), (b) Zn(II), (c) Cd(II) and (d) Pb(II) on LTA zeolite with time (Initial solution concentration: 100 mg/L, adsorbent dose: 2.5 g/L, pH: Initial pH of the solution, 5.2).

**Fig. S6.** Loading of (a) Cu(II), (b) Zn(II), (c) Cd(II) and (d) Pb(II) on LTA zeolite with time (Initial solution concentration: 150 mg/L, adsorbent dose: 2.5 g/L, pH: Initial pH of the solution, 5.2).

Kinetic parameters of intra-particle diffusion model for NaP and LTA zeolite are presented in Table S4.

**Table S4.**

Kinetic parameters of intra-particle diffusion model (IPD) for NaP and LTA zeolite.

| Adsorbent | Model | Model Parameters | 100 mg/L | | | |  | 150 mg/L | | | |
| --- | --- | --- | --- | --- | --- | --- | --- | --- | --- | --- | --- |
|  |  |  | Cu | Zn | Cd | Pb |  | Cu | Zn | Cd | Pb |
| NaP |  | q_e_ (mg/g) | 30.52 | 5.94 | 23.52 | 37.44 |  | 32.76 | 2.00 | 23.56 | 56.02 |
|  | IPD | k_i_ | 0.341 | 0.118 | 0.489 | 0.0005 |  | 0.315 | 0 | 0.360 | 0.001 |
|  |  | C | 20.21 | 2.62 | 9.73 | 37.07 |  | 22.73 | 2.794 | 12.35 | 56.00 |
|  |  | R^2^ | 0.907 | 0.707 | 0.787 | 1 |  | 0.961 | 0.491 | 0.902 | 1 |
| LTA |  | q_e_ (mg/g) | 37.58 | 33.96 | 36.79 | 37.07 |  | 50.25 | 18.52 | 41.30 | 56.01 |
|  | IPD | k_i_ | 0.087 | 0.319 | 0.143 | 0.001 |  | 0.339 | 0.093 | 0.263 | 0.001 |
|  |  | C | 35.51 | 25.32 | 33.27 | 37.06 |  | 40.96 | 16.00 | 33.89 | 55.97 |
|  |  | R^2^ | 0.989 | 0.919 | 0.973 | 1 |  | 0.968 | 0.977 | 0.967 | 1 |

Intra-particle diffusion kinetic model plots for (a) Cu(II), (b) Zn(II), (c) Cd(II) and (d) Pb(II) on NaP zeolite are shown in Fig. S7 and Fig. S8.

**Fig. S7.** Intra-particle diffusion kinetic model plots for (a) Cu(II), (b) Zn(II), (c) Cd(II) and (d) Pb(II) on NaP zeolite (Initial solution concentration: 100 mg/L, adsorbent dose: 2.5 g/L, pH: Initial pH of the solution, 5.2).

**Fig. S8.** Intra-particle diffusion kinetic model plots for (a) Cu(II), (b) Zn(II), (c) Cd(II) and (d) Pb(II) on LTA zeolite (Initial solution concentration: 150 mg/L, adsorbent dose: 2.5 g/L, pH: Initial pH of the solution, 5.2).

Boyd kinetic model plots for (a) Cu(II), (b) Zn(II), (c) Cd(II) and (d) Pb(II) on NaP zeolite are shown in Fig. S9 and Fig. S10.

**Fig. S9.** Boyd kinetic model plots for a(a) Cu(II), (b) Zn(II), (c) Cd(II) and (d) Pb(II) on NaP zeolite (Initial solution concentration: 100 mg/L, adsorbent dose: 2.5 g/L, pH: Initial pH of the solution, 5.2).

**Fig. S10.** Boyd kinetic model plots for (a) Cu(II), (b) Zn(II), (c) Cd(II) and (d) Pb(II) on LTA zeolite (Initial solution concentration: 150 mg/L, adsorbent dose: 2.5 g/L, pH: Initial pH of the solution, 5.2)..

**References**

[1] Lin, Z., Yuan, P., Yue, Y., Bai, Z., Zhu, H., Wang, T., Bao, X., 2020. Selective adsorption of Co (II)/Mn (II) by zeolites from purified terephthalic acid wastewater containing dissolved aromatic organic compounds and metal ions. Sci. Total Environ., pp. 134287. https://doi.org/10.1016/j.scitotenv.2019.134287.

[2] Langmuir, I., 1918. The adsorption of gases on plane surfaces of glass, mica and platinum. J. Am. Chem. Soc. 9, pp. 1361-1403. <https://doi.org/10.1021/ja02242a004>.

[3] Repo, E., 2011. EDTA-and DTPA-Functionalized Silica Gel and Chitosan Adsorbents for the Removal of Heavy Metals from Aqueous Solutions. Lappeenranta University of Technology, Laboratory of Green Chemistry.

[4] Dubinin, M.M., Zaverina, E.D., Radushkevich, L.V., 1947. Sorption and structure of active carbons. I. Adsorption of organic vapors. Zhurnal Fizicheskoi Khimii 21, 151-162.

[5] Tavlieva, M.P., Genieva, S.D., Georgieva, V.G., Vlaev, L.T., 2013. Kinetic study of brilliant green adsorption from aqueous solution onto white rice husk ash. J. Colloid Interface Sci. 409, 112-122. <https://doi.org/10.1016/j.jcis.2013.07.052>.

[6] Cherian, C., Siddiqua, S., 2021. Engineering and environmental evaluation for utilization of recycled pulp mill fly ash as binder in sustainable road construction. J. Clean. Prod. 298, 126758. <https://doi.org/10.1016/j.jclepro.2021.126758>.

[7] Munawar, M.A., Khoja, A.H., Hassan, M., Liaquat, R., Naqvi, S.R., Mehran, M.T., Abdullah, A., Saleem, F., 2021. Biomass ash characterization, fusion analysis and its application in catalytic decomposition of methane. Fuel 285, 119107. https://doi.org/10.1016/j.fuel.2020.119107.

[8] Bohra, S., Kundu, D., Naskar, M.K., 2014. One-pot synthesis of NaA and NaP zeolite powders using agro-waste material and other low-cost organic-free precursors. Ceram. Int. 1, pp.1229-1234. <https://doi.org/10.1016/j.ceramint.2013.06.001>.

[9] Zhang, Y., Chen, Y., Kang, W., Han, H., Song, H., Zhang, C., Wang, H., Yang, X., Gong, X., Zhai, C., 2020. Excellent adsorption of Zn (II) using NaP zeolite adsorbent synthesized from coal fly ash via stage treatment. J. Clean. Prod., pp.120736. https://doi.org/10. 1016/j.jclepro.2020.120736.

[10] Wang, Y., Du, T., Jia, H., Qiu, Z., Song, Y., 2018. Synthesis, characterization and CO_2_ adsorption of NaA, NaX and NaZSM-5 from rice husk ash. Solid State Sciences, pp. 24-33. <https://doi.org/10.1016/j.solidstatesciences.2018.10.003>.

[11] Zhang, Y., Han, H., Wang, X., Zhang, M., Chen, Y., Zhai, C., Song, H., Deng, J., Sun, J., Zhang, C., 2021. Utilization of NaP zeolite synthesized with different silicon species and NaAlO_2_ from coal fly ash for the adsorption of Rhodamine B. J. Hazard. Mater., 125627. https://doi.org/10.1016/j.jhazmat.2021.125627.

[12] Bohra, S., Kundu, D., Naskar, M.K., 2013. Synthesis of cashew nut-like zeolite NaP powders using agro-waste material as silica source. Mater Lett, pp.182-185. https://doi.org/10. 1016/j. matlet.2013.04.080.

[13] Muriithi, G.N., Petrik, L.F., Doucet, F.J., 2020. Synthesis, characterisation and CO_2_ adsorption potential of NaA and NaX zeolites and hydrotalcite obtained from the same coal fly ash. Journal of CO_2_ Utilization, pp. 220-230. <https://doi.org/10.1016/j.jcou.2019.11.016>.7.

[14] Azizian, S., 2004. Kinetic models of sorption: a theoretical analysis. J. Colloid Interface Sci. 1, 47-52. <https://doi.org/10.1016/j.jcis.2004.03.048>.
